# Mitochondrial Differentiation during Spermatogenesis: Lessons from *Drosophila melanogaster*

**DOI:** 10.3390/ijms25073980

**Published:** 2024-04-03

**Authors:** Viktor Vedelek, Ferenc Jankovics, János Zádori, Rita Sinka

**Affiliations:** 1 Department of Genetics, Faculty of Science and Informatics, University of Szeged, 6726 Szeged, Hungary; 2 Institute of Genetics, HUN-REN Biological Research Centre, 6726 Szeged, Hungary; jankovic@brc.hu; 3 Department of Medical Biology, Albert Szent-Györgyi Medical Centre, University of Szeged, 6720 Szeged, Hungary; 4 Institute of Reproductive Medicine, Albert Szent-Györgyi Medical Centre, University of Szeged, 6723 Szeged, Hungary; zadori.janos@med.u-szeged.hu

**Keywords:** mitochondria, mitochondrial differentiation, spermatogenesis, *Drosophila melanogaster*, testis, nebenkern, paracristalline material

## Abstract

Numerous diseases can arise as a consequence of mitochondrial malfunction. Hence, there is a significant focus on studying the role of mitochondria in cancer, ageing, neurodegenerative diseases, and the field of developmental biology. Mitochondria could exist as discrete organelles in the cell; however, they have the ability to fuse, resulting in the formation of interconnected reticular structures. The dynamic changes between these forms correlate with mitochondrial function and mitochondrial health, and consequently, there is a significant scientific interest in uncovering the specific molecular constituents that govern these transitions. Moreover, the specialized mitochondria display a wide array of variable morphologies in their cristae formations. These inner mitochondrial structures are closely associated with the specific functions performed by the mitochondria. In multiple cases, the presence of mitochondrial dysfunction has been linked to male sterility, as it has been observed to cause a range of abnormal spermatogenesis and sperm phenotypes in different species. This review aims to elucidate the dynamic alterations and functions of mitochondria in germ cell development during the spermatogenesis of *Drosophila melanogaster*.

## 1. Introduction

The mitochondrion is a double membrane-bounded cell organelle that contains several copies of mitochondrial DNA (mtDNA). Despite its own genetic material, the mitochondrial function is mainly completed by proteins encoded in the nuclear genome. The most prominent function attributed to mitochondria is the production of ATP through oxidative phosphorylation (OXPHOS). However, these organelles also function as calcium storage units, exert significant influence on reactive oxygen species (ROS) signaling, contribute to heat generation, facilitate the synthesis of steroids, regulate apoptotic cell death, and participate in various other biological processes, including the provision of energy for the motility of sperms. The outer mitochondrial membrane isolates the mitochondrion from the cytoplasm; however, it is highly permeable to many cytosolic compounds compared to the inner membrane. Due to its folding and the formation of the mitochondrial cristae, the inner membrane exhibits a considerably larger surface area than the outer membrane. The inner membrane contains various transmembrane and membrane-bound proteins, including the elements of the electron transport chain, the ATP synthase, and transporter proteins. The lumen of the inner membrane is the mitochondrial matrix, which contains the mitochondrial DNA and ribosomes. An example of mitochondrial structural versatility is observable in the steroid-producing cells, where the characteristic mitochondrial cristae structure forms [1]. Mitochondria in spermatocytes also show a special cristae structure [1,2].

**Figure 1:**
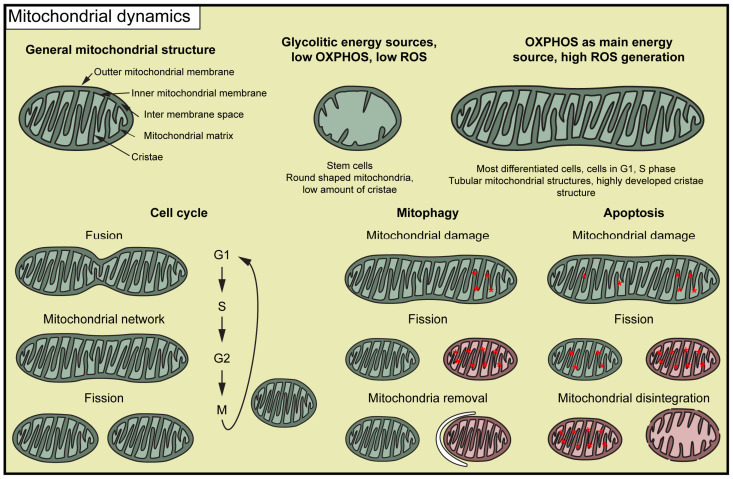
Mitochondrial structure and dynamics. The red asterisks represent mitochondrial damage. Red colouring represents malfunctioning mitochondria.

The dynamical changes between mitochondrial morphology and function are observable during the cell cycle [3]. The initially fragmented mitochondria start to form a mitochondrial network at the end of the G1 and S phases. This is believed to ensure the energy requirements for the replication of DNA and cell cycle progression. The mitochondrial network becomes fragmented during the M phase; therefore, they could be evenly distributed between the daughter cells [3]. Mitochondrial segmentation also occurs under stress conditions, which can trigger mitophagy or apoptosis [4,5] (Figure 1).

**Figure 2:**
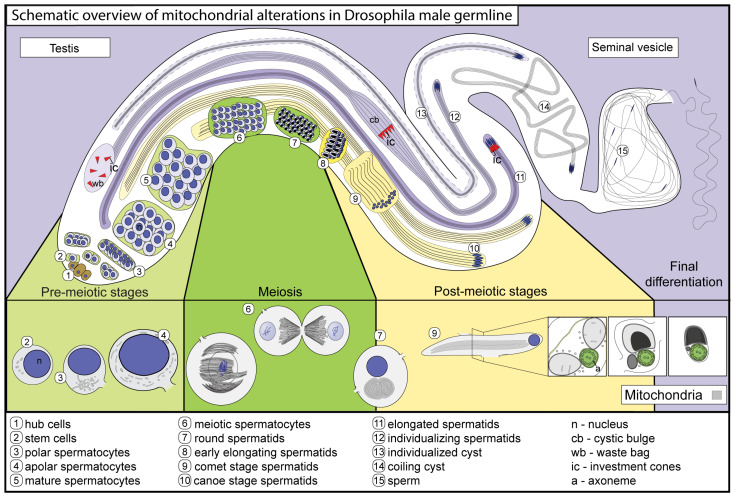
Schematic representation of the major stages of spermatogenesis and the structural changes in mitochondria in *Drosophila melanogaster* testes. The upper part of the image represents a Drosophila testis, the stem cell niche at the apical tip faces to the left, and mature sperm are present on the right side. The lower part of the image exemplifies the notable changes in mitochondria in developing germ cells. The following colour code applies to the figures that follow: the light green background represents pre-meiotic stages, green represents meiotic stages, yellow stands for early post-meiotic stages, and purple represents late post-meiotic stages and the matured sperm.

Mitochondrial activity in spermatogenesis and in sperm function is essential, where the differentiation of mitochondria during spermatogenesis includes changes in morphology and molecular components [6]. During *Drosophila melanogaster* spermatogenesis, intensive mitochondrial dynamics are observable throughout various stages of development, starting from the initial steps of germline stem cell (GSC) differentiation to the final differentiation of sperm cells (Figure 2). During mammalian spermatogenesis, the mitochondrial structure undergoes a significant reorganization that is unique to each stage of meiosis [7,8]. Mitochondrial fission and fusion events, along with changes in the cristae structure, are observed in spermatogonia and spermatocytes, resembling the characteristics found in somatic cells [2,7]. Mitochondrial differentiation in Drosophila spermatids results in one of the most extreme mitochondrial forms observable in nature: where two mitochondrial derivatives elongate with the spermatid tail, reaching approximately 1.8 mm in length. In elongating Drosophila spermatids, two tubular mitochondrial derivatives run along the entire length of the tail, modulating the elongation process of the cyst [9]. In the mature sperm, one of the derivatives is filled with electron-dense paracrystalline material, while the other derivative loses the majority of its volume, yet is present in the giant mature sperm tail [10,11]. These structural and functional alternations are spectacular in many aspects; nevertheless, the molecular and functional differences between the two mitochondrial derivatives are barely known. In this review, we summarize the changes in mitochondrial morphology during Drosophila spermatogenesis and highlight the molecular components and the structural changes required to establish the specialized mitochondria of the sperm.

## 2. Stem Cell Maintenance and Spermatocyte Differentiation

**Figure 3:**
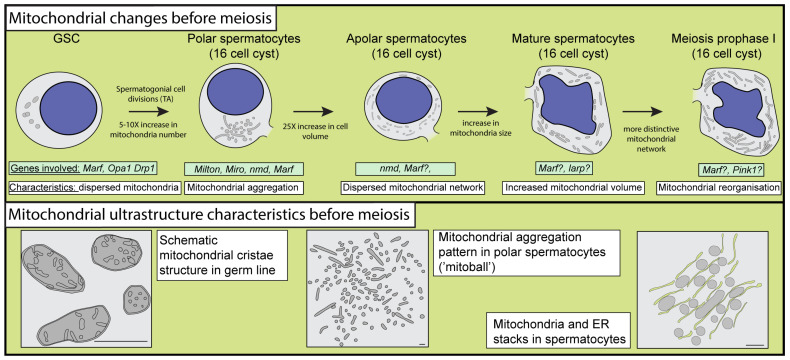
Schematic illustrations of the changes in mitochondrial morphology and distribution before meiosis in Drosophila. The mitochondrial distribution in germline stem cells and polar, apolar, and mature spermatocytes is highlighted in the upper section. The bottom section represents the mitochondrial organization in pre-meiotic germ cells. Question marks represent the potential role of the genes in the developmental step.

The stem cell niche is maintained by the somatic hub cells at the apical tip of the Drosophila testis. In this niche, the GSCs and cyst stem cells (CySCs) reside in close proximity to the hub cells. Asymmetric GSC divisions generate two cells: one of the daughter cells remains in the niche and functions as a GSC; meanwhile, the other one becomes a spermatogonial cell committed to further differentiation. The freshly differentiated spermatogonia is consorted by two somatic cyst cells, which later establish a single cyst: the cyst cells encapsulate the developing germ cells during spermatogenesis. Following their exit from the stem cell niche, the spermatogonia proceed to undergo a series of four mitotic divisions, commonly referred to as transit amplification (TA) divisions. During these divisions, the size of the spermatogonia does not change considerably, and the spermatocytes remain connected through ring canals (Figure 3) [10].

In Drosophila testes, mitochondrial fusion is required for stem cell maintenance, while mitochondrial fission is needed for germ cell differentiation [12,13,14]. The Drosophila mitofusins Marf and Optic Atrophy 1 (Opa1) regulate the fusion of the outer and inner mitochondrial membranes and are necessary for GSC maintenance [12]. In the absence of these proteins, mitochondrial activity and general mitochondrial health are decreased, as this malfunction of mitochondria results in decreased fatty acid oxidation (FAO) and fatty acid (FA) enrichment in the cytosol of GSCs (Figure 3).

The maintenance of GSCs relies heavily on maintaining a delicate balance between mitochondrial fusion and fission. This notion is further supported by the findings that silencing *Dynamin-related protein 1* (*Drp1*), a dynamin-like GTPase crucial for mitochondrial fission, in GSCs resulted in a higher rate of germ cell loss. This could be due to premature differentiation in the *Drp1* mutant germline, where the elevated amount of ROS could contribute to the loss of GSCs [14]. It was shown that elevated ROS levels and disturbance in the antioxidants in GSCs manifest in the differentiation and loss of GSCs [15]. On the other hand, a reduction in ROS levels by Keap1 RNAi or antioxidants leads to the facilitation of GSC growth. The elevated ROS levels in GSCs result in the activation of EGFR signaling in the cyst cells, where TOR signaling regulates autophagy in maintaining the stem cells and lipid homeostasis. Additionally, mitochondrial differentiation is also crucial for somatic cyst cell differentiation in Drosophila testes [16]. The role of ROS is also known in mammalian spermatogenesis, as it is required for spermatogonial differentiation [7,17,18].

The TA divisions result in a 16-cell cyst, containing the primary spermatocytes. The young spermatocytes are called polar spermatocytes, where their nucleus is acentric, and the mitochondria aggregate at the opposite pole. During differentiation, the spermatocytes’ volume increases approximately 20–25× and they become apolar spermatocytes. In these cells, the nucleus has a central position, DNA condensation is minimal, and intensive transcription occurs. The vast majority of the transcripts that are required for later development are produced in primary spermatocytes. During normal spermatogenesis, approximately 30% of the spermatogonial cysts undergo germ cell death (GCD). This mechanism is considered programmed necrosis, which is regulated by p53 and the Drosophila ortholog of caspase-9 [19]. It has been shown that p53-dependent necrosis is conserved from invertebrates to vertebrates to maintain a healthy GSC number [20]. GCD has both lysosomal and mitochondrial components. One of the main mitochondrial components of GCD is the apoptosis inhibitor HTRA2-related serine protease (*HtrA2*), which is enriched in testes [21,22]. *HtrA2* shows genetic interaction with *PTEN-induced putative kinase 1* (*Pink1*), a component of the *Pink1/Parkin* pathway, which is known to be important for mitochondrial quality control and mitophagy not just in the germline, but in somatic cells [23,24] (Figure 3). The Pink1 kinase is capable of the phosphorylation of Parkin on the mitochondrial surface, which in turn could act as an E3 ubiquitin ligase, and regulate the turnover of mitochondrial proteins and induce mitophagy.

In polar spermatocytes during the TA divisions of spermatogonia, mitochondria dynamically aggregate, forming a mitoball structure [10,12]. This specific aggregation of mitochondria could serve as a mitochondrial quality checkpoint; however, experimental evidence shows no mitophagy in this state [25]. Mitochondrial aggregation has also been described in ovaries, suggesting that the fusion of mitochondria could be connected with an elevated energy demand, such as intensive growth or the reorganization of cellular organelles during both oogenesis and spermatogenesis [25,26,27]. In polar spermatocytes, the formation of mitoballs requires Milton-dependent mitochondrial trafficking [25]. Milton is a myosin-binding protein that exhibits microtubule motor activity and it is associated with the Pink1/Parkin interactor Miro, which is a mitochondrial Rho GTPase that performs calcium and magnesium binding [9,28,29]. The Milton/Miro complex creates a link to kinesin motors, enabling Ca^++^-dependent mitochondrial transport [30].

In the apolar spermatocytes, the mitochondria disperse in the cytosol and the mitochondrial number increases [10,11]. In a mature 16-cell cyst, mitochondrial structures become interconnected with the endoplasmic reticulum and the size of mitochondria is slightly larger than in the apolar spermatocytes [10,25]. Drp1 is required for normal mitochondrial distribution at this stage [28].The presence of a more structured mitochondrial organization in mature spermatocytes is a hallmark of the initiation of meiotic divisions. The arrangement of mitochondria in mature spermatocytes undergoes a significant reorganization before the commencement of meiotic divisions. This reorganization manifests in the formation of parallel structures, characterized by the alignment of rod-like mitochondria in a parallel fashion [10].

## 3. Meiosis

The Drosophila mature spermatocytes enter meiosis simultaneously, and the meiotic cell divisions result in a 64-cell cyst where the spermatids are still interconnected with ring canals. There is an intimate connection between the mitochondria and the microtubules during meiosis [10,31]. The anchoring of the mitochondria to the microtubules might be mediated by the Ifc, a sphingosine delta 4 desaturase enzyme [32]. The *ifc* mutants fail to enter meiotic cell divisions; therefore, *ifc* might act as a checkpoint of mitochondrial organization [33]. It seems that the mitochondria’s organization also affects the proper central spindle formation [34]. In *mitoshell* mutant spermatocytes, abnormal mitochondrial aggregation occurs, which results in defective spindle organization [34]. Since a variety of mitochondrial markers are available for tracing mitochondrial differentiation during meiosis, it becomes easier to investigate the mitochondrial fate during the divisions (bb8^N100aa^-GFP, S-lap3-GFP, Sprn, anti-Atp5alpha, CG10748-GFP, Knon-GFP, anti-Merlin) [35,36,37,38,39]. With the above-mentioned fluorescent mitochondrial markers, we can observe a barrel-shaped mitochondrial network around the nuclear region of cells in metaphase, which later has a cylindrical shape in anaphase and telophase. Interestingly, these mitochondrial structures are not present in a punctuated pattern: they seem to have a tubular structure. This structure remains organized parallel to the spindle. This is reinforced by Tates’s observations that the mitochondrial structures in meiosis I show a rod shape with a diameter of 0.58 μm and a length up to 8 μm, and line up parallel in proximity to the nuclear membrane [10,11,40]. It is important to notice that the formation of these rod-shaped mitochondria presumably is mediated by the Marf mitofusin. There is a correlation between the appearance of fused mitochondria and *Marf* expression in early developmental stages [41]. During meiosis I telophase, mitochondria remain in the central part of the cell, and as cytokinesis progresses, they form a butterfly-like pattern [10,42,43]. During meiosis II, the process repeats itself; the mitochondria aggregate around the nuclei, then remain in the axis of the cytokinetic furrow, which halves them.

## 4. Early Spermatids

**Figure 4:**
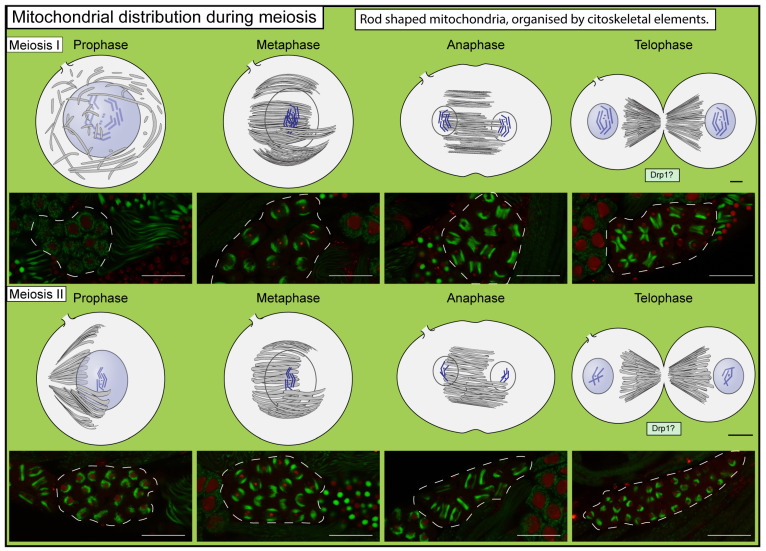
Schematic modeling of mitochondrial morphology changes during meiosis in Drosophila. Microscopic images represent each stage (the top section represents the first meiotic division, and the bottom section represents the second meiotic division), wherein mitochondria were visualized with CG10748-GFP (green) and nuclei were visualized with His2Av-mRFP1 (red) [82,83], and live samples were imaged using an Olympus Fw10 confocal microscope. Dashed lines highlight individual cysts. Question marks represent the potential role of Drp1 in the developmental step. On microscopic images, scale bars represent 50 μm.

**Figure 5:**
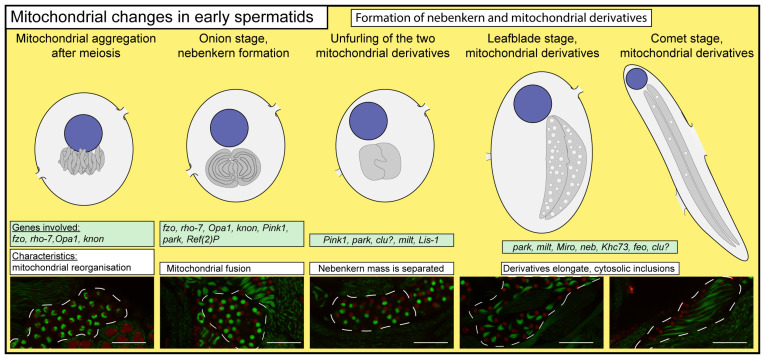
Mitochondrial changes in young spermatids in Drosophila. Images representing the formation of a nebenkern after meiosis and the mitochondrial differentiation in the early elongation stages. Schematic drawings were made based on microscopic observations and data from the literature. On microscopic images, mitochondria were visualized using CG10748-GFP (green) and nuclei were visualized using His2Av-mRFP1 (red) [82,83]; live samples were imaged using an Olympus Fw10 confocal microscope. Dashed lines highlight individual cysts. Question marks represent the potential role of the genes in the developmental step. Scale bars represent 50 μm.

After the process of meiosis, the mitochondria of the round spermatids aggregate and fuse to create the nebenkern structure. This structure is composed of two mitochondrial derivatives that intertwine with each other, resembling an onion shape and occupying a size similar to that of the haploid nucleus (Figure 4 and Figure 5). Nebenkern formation is fundamental for the developing spermatids; any disturbance in nebenkern formation has detrimental consequences in later developmental stages during elongation and individualization.

In *twine* and *mtsh* mutants, the second meiotic division is absent or abnormal; however, a nebenkern forms and the mutant cyst starts to elongate [34,44]. This suggests that the mitochondrial aggregation is programmed and the necessary factors for nebenkern formation are present at the end of the last cytokinesis. The visualization of rod-shaped mitochondrial dynamics during cell divisions highlights the potential role of these structures in the equitable distribution of mitochondrial mass. The elongated, rod-shaped mitochondria of a larger size may serve as optimal precursors for nebenkern formation and can be preserved throughout the process of cell division. This hypothesis is supported by the observation of Drp1 mutants, in which the mitochondria fail to properly segregate at the end of meiotic telophase. Instead, these mitochondria traverse the cytoplasmic bridges, leading to the formation of dispersed masses of mitochondria (Figure 4). Consequently, an abnormal ratio between the mitochondria and nuclei is observed in *Drp1* mutants, indicating the involvement of Drp1 in the division of rod-like mitochondria during cytokinesis [28].

There are three observable stages of mitochondrial reorganization before the formation of the onion-stage nebenkern [10]. In the coalescence stage, the mitochondria aggregate to the side of the nucleus. At this stage, it is most likely that the rod-like mitochondria undergo bending and clustering, which is potentially driven by microtubules. The necessity of microtubules for nebenkern formation is debated, but colchicine treatment could result in small and dispersed mitochondrial derivatives [10,45]. During the agglomeration stage, the mitochondria form a single mass and start to fuse. In the clew stage, the mitochondrial mass becomes spherical and positioned near the nucleus [10]. After the clew stage, two mitochondrial derivatives are tightly intertwined with each other within the onion-stage nebenkern.

The first gene described related to mitochondrial dynamics was *fuzzy onions* (*fzo*), as it is required for mitochondrial fusion during nebenkern formation in Drosophila spermatids [46] (Figure 5). In *fzo* mutants, the nebenkern formation is defective: the mitochondria are unable to aggregate and fuse properly and instead of the establishment of the two mitochondrial derivatives, they form multiple smaller derivatives [46]. In addition to *Fzo*, *Rhomboid-7* (*Rho-7*), an intramembrane serine protease, and *Opa1*, a dynamin-related GTPase, are also required for membrane fusion and nebenkern formation [47]. *Rho-7* acts upstream of *Pink1* in the Pink1/Parkin pathway [48] (Figure 5).

For the formation and shaping of the onion-like membrane layers of the nebenkern, the testis-specific Knon is essential [38]. Knon is an ATP synthase subunit, a paralog to ATPsyn5D. In addition to Knon, ATPsynCF6L and ATPsynGL subunits of ATPase are also involved in proper mitochondrial development in the testis [38].

In mammalian meiotic spermatocytes, the mitochondria also show dynamic reorganization fusion and fission during divisions, but after meiosis, they undergo fission [8,49]. This is required for proper mitochondrial sheath formation, and most probably for removing the excess or malfunctioning mitochondria to residual bodies. Whether the mechanism behind the elongation of mitochondria present in the midpiece involves mitochondrial fusion is still debated [49,50].

In Drosophila, the fusion of nebenkerns is a typical phenotype of many meiotic cytokinesis mutants (Deterin, anilin, tBRD-1, tBRD-2, Hbs1, pelota, fumble, doublefault, Dhc64C, Klp61F, Khc-73, pavarotti, Nebbish, Fascetto, asunder, fws); this phenotype manifest in multiple nuclei attached to a single larger nebenkern [9,43,51,52,53,54,55,56,57,58,59]. Cytochalasin B treatment has similar severe effects, suggesting that mitochondrial aggregation is most likely regulated by meiotic divisions through microtubules and motor proteins [60]. La-related protein (Larp) mutants show similar cytokinesis phenotypes. Larp is localized to the mitochondria during meiosis II and plays a central role in partitioning the cell organelles [31,61,62]. Based on the molecular function of Larp, as a translation regulator poly(A)-binding protein, it could participate in mitochondrial protein synthesis [61]. The phosphorylation of Larp by Pink1 results in the silencing of local protein biosynthesis on the mitochondria [63]. As previously mentioned, in the primary spermatocytes, the mitochondria grow both in number and size, which most probably heavily relies on the local protein synthesis machinery. In ovaries, Larp is recruited by the mitochondrial surface protein, Mdi, enhancing local protein synthesis and mitochondrial biogenesis [64]. A similar function is also plausible in testes. Nevertheless, at the onset of meiosis, when the chromatin is condensed, and when mitochondrial positioning and interaction with microtubules are becoming crucial, the protein synthesis at the mitochondria needs to be silenced, which could be achievable through Pink1–Larp interactions. The cytosolic dynein-1 motor complex regulator (Lis-1) mutants and Dynein light-chain 90F (Dlc90F) mutants do not show severe cytokinesis defects, but they exhibit an interesting phenotype. Occasionally (~15%, ~3%, respectively), an additional nebenkern-like derivative forms next to a single nucleus [62]. A similar phenotype is observable in the dynein subunit wampa (wam) mutants, where cytokinesis defects result in abnormal nebenkern formation and smaller mitochondrial derivatives [42]. These results suggest the important role of microtubule-associated proteins in mitochondrial differentiation during and after meiosis.

The formation and maintenance of the two derivatives from the nebenkern most likely rely on the Pink1/Parkin pathway [65,66]. In both Pink1 and Parkin mutants, the nebenkern becomes vacuolated and only a single mitochondrial derivative forms during elongation [65,67,68,69]. *Pink1* and *fzo* show genetic interaction; in double mutants, the *Pink1* mutant phenotype manifests [66]. Pink1/Parkin is likely to promote the degradation of Fzo to restrict its function and avoid the fusion of the two mitochondrial derivatives. The Pink1/Parkin pathway may be subject to the influence of additional genes, which could impact the process of mitochondrial differentiation in spermatids. The mutants of Drosophila orthologs of the autophagy gene *p62*, *ref(2)P* exhibit mitochondrial defects in round spermatids, and it is required for mitochondrial aggregation and for the phenotypic suppression of the *pink1* mutant phenotype. [70]. The *PARK7* Drosophila orthologs also play a role in spermatogenesis, namely *DJ-1alpha* and *Dj-1beta*. *Dj-1alpha* exhibits testis-specific enrichment, while *DJ-1beta* has an expression minimum in testes. In spite of this, the double knockout of these genes results in male sterility and mitochondrial abnormalities in spermatids [71]. There are four uncoupling protein 4 (*UCP4*) genes in Drosophila that encode mitochondrial carrier proteins and show genetic interaction with the Pink1/Parkin pathway. When overexpressed, the *UCP4A*, *UCP4B*, and *UCP4C* genes are capable of rescuing the Pink1 male-sterile phenotype. Interestingly, *UCP4B* and *UCP4C* exhibit a testis-enriched expression profile [72]. Overexpression of the testis-enriched Neddylase subunit APP-BP1 also suppresses Pink1 RNAi-induced male sterility [73]. Neddylation of Pink1 increases its stability and improves its activity [73]. The overexpression of the testis-enriched *Fer3HCH* gene is also capable of rescuing the mutant Pink1 phenotype in neurons; however, its role was not studied in testes [74,75]. *Pink1* and *Parkin* show genetic interaction with *Miro*, which is essential for mitochondrial homeostasis and microtubule-based mitochondrial transport in neurons [29]. The mitochondrial E3 ligase *Mul1* also exhibits testis-specific enrichment and it has a function in the ubiquitination of *Marf*; therefore, it is capable of compensating for the loss of Pink1/Parkin. However, its function during spermatogenesis has not been studied [76].

In the Pink1 pathway, downstream of Pink1, the HtrA2 inhibitor of apoptosis is present. In *HtrA2* mutants, the major mitochondrial derivative does not show morphological abnormalities; however, the *HtrA2* mutant has an abnormal spermatid individualization after elongation [77]. The testis-enriched *dYME1L* is an ortholog of *YME1*, an AAA protease localized at the mitochondrial inner membrane, responsible for mitochondrial quality control. *HtrA2* mutation can suppress retinal degeneration in *dYME1L*-deficient flies. The loss of dYME1L causes an uncharacterized male sterility [78]. During the early mid-elongation phase of spermatids in *parkin* mutants, the mitochondrial derivatives show an uneven distribution along their longitudinal axis, similar to that observed in *clueless* mutants [69,79]. Clueless is a conserved cytosolic protein that interacts with the Pink1/Parkin complex [80], and it promotes TER94 /VCP-mediated Marf degradation [81]. Taken together, the Pink1/Parkin pathway plays a central role in the regulation of mitochondrial function and morphogenesis. One of their main functions during spermatid differentiation is probably the elimination of the Fzo and Marf mitofusins after nebenkern formation.

## 5. Spermatid Elongation

During the formation of the nebenkern and the mitochondrial derivatives, the cysts become polarized and start to elongate. At the beginning of cyst elongation, the nebenkern is anchored to the nuclei; this is mediated by Milton and Lis-1 [28,62]. When the 64-round spermatids start to elongate, the mitochondria enter first the leaf-blade stage and later the comet stage. In the early stages of the elongating cyst, the nebenkerns unfurl to establish the two mitochondrial derivatives in each spermatid, while the axonemes also start to elongate (Figure 5). As the spermatids differentiate, the nebenkern unfurls, and establishes two leaf-blade-shaped mitochondrial masses. At this stage, the mitochondrial derivatives still have interconnected membranes and they have characteristic cytosolic inclusions, which are also visible with phase-contrast microscopy [10,11].

During elongation, the mitochondrial derivatives completely separate, the cytosolic inclusions gradually vanish, and a connection with the ER-derived axial membrane is established. Next, as the spermatids elongate, they enter the comet stage, where the round nucleus has a comet-like tail. At the comet stage, the two mitochondrial derivatives have similar volumes and elongate at almost the entire length of the developing spermatids.

**Figure 6:**
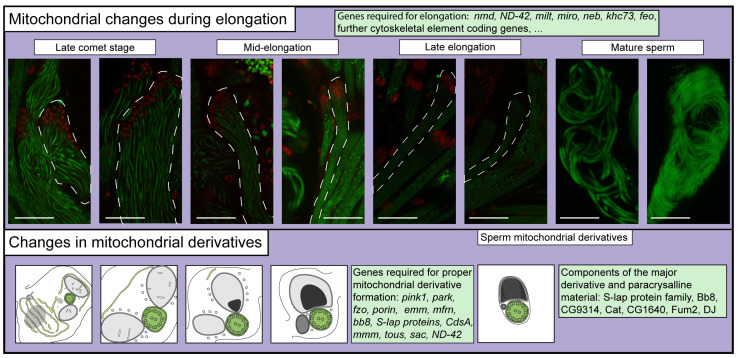
Microscopic images representing the multiple elongation stages of Drosophila spermatids, wherein mitochondria were visualized with CG10748-GFP (green), in mature sperm with Dj-GFP (green), and nuclei were visualized using His2Av-mRFP1 (red) [82,83,100]. Dashed lines highlight individual cysts. Scale bars represent 50 μm.

During the elongation, the cyst cells also differentiate: one becomes the head cyst cell, while the other one becomes the tail cyst cell. After the comet stage, spermatids continue to elongate, while their nucleus also starts to condensate, as the histone–protamine transition takes place. The initially similar mitochondrial derivatives start to differentiate during elongation: in one of them a paracrystalline material accumulates, while the other one loses its volume (Figure 6). The elongation of the spermatids is mainly driven by the mitochondrial derivatives, the cytosolic microtubule array, and an actin and spectrin-rich elongation complex at the basal end of the cyst [9,84]. The elongation occurs in a growth zone at the basal end, close to the elongation complex, while the already elongated parts are becoming stable [9]. During spermatid elongation, Milton/Miro localizes to the basal end of the cyst and enables the sliding of cytosolic microtubules, which, in this way, allows the growth of the mitochondria. In the already-elongated regions, the microtubules are cross-linked by Nebbish, Khc73, and Fascetto, and establish an array to stabilize the mitochondria [9]. In the absence of normal mitochondrial derivatives, elongation is abnormal.

In the absence of the ATPase *nmd*, the number of mitochondria is decreased in spermatocytes, which results in abnormal development in later stages: the nebenkern and consequently the mitochondrial derivatives become smaller, leading to spermatid elongation defects [9]. Similarly, in NADH dehydrogenase (ubiquinone) 42 kDa subunit (ND-42) (Mitochondrial Respiratory Complex I) knockdown flies, mitochondrial derivatives do not form properly, resulting in abnormalities in elongating cysts [85].

Several lines of evidence suggest that the cytosolic MT array is nucleated on the surface of the mitochondria and probably contributes to their elongation during cyst elongation [9,86]. In contrast to the impact of mitochondrial elongation defects on cyst elongation, axonemal microtubules are not essential for the elongation process [35,87]. Microtubules around the mitochondrial derivatives are responsible for the elongation of the mitochondria and consequently the elongation of the axoneme [9,86]. The elongation of cysts and the spermatozoa is independent of the axoneme formation; it is related to the function of the mitochondrial derivatives and the accessory microtubules around them [9,87]. Cnn is required for male meiosis and assembly of the flagellar axoneme [86]. It was reported that a testis-specific isoform of Cnn (CnnT) and Mzt1 localizes to the nebenkern in round spermatids and possibly recruits microtubules to the surface of the mitochondria and converts mitochondria to a non-centrosomal Microtubule Organizing Center (ncMTOC) [86,88]. The testis-specific γ-TuRC members t-Grip84, t-Grip91, t-Grip128, and Mzt1 have been shown to localize to the basal body and are likely together with CnnT to be part of the ncMTOCs found on the mitochondrial surface of the elongated spermatids [86,88,89]. The dynein-related *tous*, *mmm*, and *sac* genes also have an impact on the proper cyst elongation by influencing the accessory microtubule organization around the mitochondrial derivatives [90].

For proper elongation and individualization, lipid biosynthesis is also necessary [91,92]. The onion-stage nebenkern contains approximately 30% of the lipids that are necessary for the fully elongated derivatives [93]. Mutation in CdsA, a CDP–diacylglycerol synthase, results in membrane overgrowth, abnormal mitochondrial derivatives in elongated spermatids, and individualization defects [94].

As the cyst matures, the two elongated mitochondrial derivatives start to differentiate: the major one starts to accumulate paracrystalline material, while the minor one starts to lose its volume. During elongation, the ER-derived axial membrane (axonemal sheath) forms a connection with the developing mitochondrial derivatives. This contact site is the origin of paracrystalline material accumulation [10,11]. At TEM cross sections, the mitochondrial derivatives and the central two axonemal microtubules show characteristic angular positioning, which is correlated with the progress of elongation [95]. At the end of elongation, the major derivative is filled by paracrystalline material, and the minor one has much less volume [95] (Figure 6). The paracrystalline material is needed for proper mitochondrial morphogenesis; furthermore, it provides structural support for the elongated spermatids and sperms. The electron-dense paracrystalline material displays resistance to SDS, enabling its purification and the identification of its components [36]. Eight members of the sperm leucyl aminotransferase (S-Lap) protein family were found to be a major component of the paracrystalline material. However, other proteins were identified as well, including Bb8 glutamate dehydrogenase, CG9314, and Cat catalases. S-Lap proteins lost their enzymatic activity, representing a good example of the neofunctionalization of somatic genes via gene duplication [36,96]. The Bb8 mutants exhibit mitochondrial identity and paracrystalline accumulation defects after the elongation of the cysts [35,97]. In the *mitoferrin* (*mfrn*) mutants, the bulging of mitochondrial derivatives and the accumulation of paracrystalline material in both mitochondrial derivatives are also observable [75]. Paracrystalline material accumulation defects could be observed in *Merlin* mutants [39], wherein paracrystalline material accumulation occurred in multiple foci in the major mitochondrial derivative, which might be a source of an additional ectopic ER–mitochondria contact site. In *park* mutants, additional paracrystalline material accumulation is observable as well [69].

For the differentiation of mitochondrial derivatives, proper protein homeostasis is required. In *RpS3* RNAi knockdown flies, mitochondrial abnormalities are observable in the late stages of spermatogenesis, suggesting that ribosomal activity is also crucial for mitochondrial differentiation [98]. In addition to protein biosynthesis, directed degradation is also necessary for the development of mitochondrial derivatives. In the mutants of the E3 ligase subunit *ago*, severe mitochondrial defects were described, including malformed derivatives [99]. Despite the fact that the main molecular components of the paracrystalline material are known, we do not know the factors restricting its formation to only one of the mitochondrial derivatives, and the molecular mechanism behind its organization and stability remains elusive.

The Drosophila-specific *dj* and *djl* genes both show testis-specific transcript enrichment, and Dj is enriched in the flagella and major mitochondrial derivative in elongated spermatid and sperm [100,101]. Despite the well-characterized and similar expression patterns of Dj and Djl, their function has yet to be revealed. Dj-GFP is a frequently used marker for the mitochondria of the elongated spermatids and matured sperm [102] (Figure 6).

The differentiation of mitochondria throughout spermatid development is a feature that extends beyond insects. In mammalian sperm, the mitochondria also possess a mechanical function by tightly coiling around the axoneme in the midpiece. This compact coiling is made possible by the presence of disulfide bonds within the mitochondrial sheath, which provide stability [2,103]. Structural abnormalities in the mitochondrial sheath often lead to abnormal sperms [50,104,105,106,107,108].

## 6. Individualization of the Spermatids

The process of individualization follows the cyst elongation, where spermatids acquire their individual membranes, while the majority of their cytosolic components are degraded and deposited into the waste bag [109]. In the individualization process, actin-rich cytoskeletal structures, the investment cones form around the nuclei and establish the individualization complex. During individualization, the cones migrate simultaneously towards the basal direction of the cyst. As the individualization complex progresses, the cystic bulge emerges and ultimately the majority of the cytosolic content including the investment cones within the cystic bulge ends up in the waste bag. As a result of individualization, the connection between spermatids is severed and the individual membranes of the sperms are formed. With the migration of the individualization complex, a caspase cascade is triggered, which does not cause apoptotic cell death; instead, it is required for proper spermatid differentiation [110].

Before being transferred to the seminal vesicle, the storage organ of the matured sperm, the sperm coil up with the involvement of the head cyst cell [10,91].

A defective individualization phenotype may arise as a result of mitochondrial abnormalities. The morphological changes in mitochondria or an energy deficit can easily disturb the synchronous migration of actin cones, resulting in malformed cystic bulges, and the failure of individualization. Mitochondria also actively contribute to the individualization process. For the initiation of the non-apoptotic caspase cascade, the mitochondrial Cyt-C-d plays an essential role. Cyt-C-d is necessary for the activation of the drICE caspase [110]. The mitochondrial inner-membrane protein succinyl-CoA synthetase b subunit (A-S-beta) seems to change its position and localize to the outer mitochondrial membrane in elongated spermatids. It binds and activates the Cullin-3-based ubiquitin ligase complex (CRL3), which is also necessary for caspase activation [101]. This association gives a spatial restriction to the caspase activation. During individualization, both mitochondrial derivatives gain their final volume. Transmission electron micrograph cross sections have indicated “mitochondrial whorls” of the small derivative in cystic bulges [111]. It is believed that the minor derivative is sheared by the progress of actin cones, which causes the removal of the mitochondrial mass that appears in the cystic bulge [112]. The remodeling of the small derivative might be beneficial for providing energy for the migrating individualization complex. Considering the size variation of pre-individualized major mitochondrial derivatives, the individualization process also removes the excess membranes from the major derivative, resulting in a uniform shape and size for the spermatid mitochondrion for both derivatives. It seems that the major mitochondrial derivative size correlates with the amount of paracrystalline material accumulated in it, and paracrystalline material accumulation might play a role in the terminal differentiation of mitochondrial derivatives [113].

Porin, a voltage-dependent anion channel (VDAC), has a male-sterile allele which shows individualization defects. Porin and Miro play a central role in the mitochondrial Ca^++^ homeostasis at ER mitochondrial contact sites (ERMCSs) in neurons [114,115]. Porin is targeted for degradation both by Parkin and Mul1. In *porin* mutants, the size of the major mitochondrial derivative varies in mature spermatids, which may be a consequence of abnormal elongation [113]. Three Porin paralogs are present in Drosophila; Porin2, CG17139, and CG17140. While *porin* shows a ubiquitous expression pattern, all of its paralogs exhibit transcript enrichment in testes [116]. Porin and Porin2 have similar structural characteristics, and they are both capable of forming a functional ion channel. In contrast to Porin2, CG17140 and CG17139 show less homology to Porin; nevertheless, CG17140 forms a smaller channel, the functionality of which is not determined, and CG17139 is not able to operate as an ion channel [117]. The main functional difference between Porin and Porin2 is that Porin2 is voltage-independent and cation-selective, while Porin is voltage-dependent and anion-selective [118]. VDACs are known to have a variety of protein interactors, including other ion channels, dimeric tubulin, and adapter proteins [119]. Both CG1740 and CG17139 *porin* orthologs have a larger N-terminal (82 AS, 61 AS, respectively) part before the Tom40 domain than Porin and Porin2 (2 AS, 5 AS). This feature propagates the idea of the neofunctionalization of these proteins, where they could act as interaction partners, and as a platform on the mitochondrial surface during the differentiation of spermatids. Porins might play a role in the formation of the contact site of the axial membrane and mitochondria.

After individualization, the mature sperm forms. The 64 sperms coil up and are stored in the seminal vesicle. At this point, mitochondria play a dual role: the major derivative gives a structural rigidity for the extremely long sperm, and it probably provides the energy supply for the movement of the sperm [10].

## 7. Mitochondrial Energetics and Metabolism

The most studied and known metabolic shift during spermatogenesis occurs with spermatogonia differentiation. The stem cells keep their low OXPHOS activity, and meanwhile, the differentiating germ cells increase the mitochondrial activity [15,16]. Our knowledge is rather limited regarding the later developmental steps. Nevertheless, the cristae structure reflects and plays an important role in the energetic and metabolic function of mitochondria [120]. The cristae structure has an impact on respiratory chain supercomplexes and respiratory efficiency [121]. However, the characterization of the cristae structure of mitochondria in developing male gametes is surprisingly poor. Based on transmission electron micrographs, the mitochondrial cristae structure is different in spermatogonia and spermatocytes compared to somatic cyst cells, not just in Drosophila, but also during mammalian spermatogenesis [2,7,12]. In the male Drosophila germ lineage, the cristae structure is not as dense as that in a somatic cyst cell. In the germ cell line, the cristae seem to have a tubular organization, meanwhile showing a lamellar structure in cyst cells (Figure 3 and Figure 6). This type of cristae structure persists during meiosis and post-meiotic development and gradually disappears as the paracrystalline material fills the major derivative (Figure 6). The function of this specialized cristae structure observed in germ lineage has not been previously investigated; however, tubular formations can be linked to steroid biosynthesis [1]. The cristae structure also depends on the lipid composition of the inner mitochondrial membrane, where cardiolipins were described to have a role in mammalian cells [122]. The maintenance of the cristae structure requires inner-membrane proteins of the mitochondrial contact site and cristae organizing system (MICOS). Eleven Drosophila genes are predicted to be the members of the MICOS: *QIL1*, *Mitofilin*, *Mic26*-*27*, *Chchd3*, *CG12479*, *CG13564*, *CG14929*, *CG15296*, *CG41128*, *CG43327* and *CG43328*. Out of these, *CG12479*, *CG13564*, *CG14929*, *CG15296*, *CG43327*, and *CG43328* show testis-specific transcript enrichment. The role of MICOS components during Drosophila spermatogenesis has not been studied. The reduced cristae structure of the mitochondria raises the possibility that Drosophila spermatogenesis does not rely heavily on OXPHOS, as high OXPHOS activity is correlated with dense cristae structures [123]. However, the silencing of Complex I member ND-42 causes male sterility, with the phenotype manifesting post-meiotically [85]. Moreover, it is important to highlight that the OXPHOS elements and the mitochondrial electron transport chain elements are also represented by testis-specific components resulting from gene duplications [83,124,125]. We can hypothesize that the higher variety of mitochondria-associated genes in the testes allows for adapting and maintaining the OXPHOS in the specialized cells. The role of the testis-specific electron transport chain members in post-meiotic spermatogenesis has not been studied systematically. An exception is the *Cox4l* gene, the testis-specific paralog of Cytochrome c oxidase 4, which was reported to have an essential function during the late stages of spermatogenesis [126]. Moreover, the role of *knon*, *blw*, and *cyt-c-d* suggests the existence of further unidentified key elements of the electron transport chain during spermatogenesis [124,125]. Interestingly, in mice, the Coxfa4l3 electron transport chain subunit was identified as a testis-specific counterpart of the Coxfa4 subunit. The testis-specific Coxfa4l3 protein replaces the generally expressed Coxfa4 subunit after meiosis [127].

Mitochondrial function is also connected with sugar metabolism and the citrate cycle. In Drosophila, many of the genes that participate in the central metabolic pathways have paralogs that exhibit testis-specific enrichment in late-developing cysts [83,128]. Despite knowing more and more about the protein composition of the post-meiotic mitochondria, we do not know how they contribute to the energy source of Drosophila sperm. The drastic reorganization events of mitochondria during spermatogenesis could result in multiple metabolic shifts. As presented previously, a metabolic shift happens in spermatogonial differentiation in Drosophila testes, which is most probably analogous to the mammalian stem cell differentiation in the testes and brain where the differentiation is accompanied by a metabolic shift from glycolysis to OXPHOS [129,130]. However, the existence of testis-specific components of both glycolysis and OXPHOS elements suggests further shifts or complementation in later developmental stages as well. RNA sequencing has shown distinct expression patterns for testis-specific metabolic enzymes; however, additional functional studies are required to clarify the presence and role of the hypothetical transitions [83,131]. In *Drosophila hydei*, metabolic changes were described in testes of between 1-day and 10-day-old males. These changes were investigated via in vitro enzyme assays and gave an insight into potential metabolic shifts [132]. Measuring a variety of enzymes, the study concluded that during the early stages of development, there is a high level of carbohydrate oxidation and enzyme activity related to amino acid metabolism and fatty acid synthesis. In late spermiogenesis, there is a significant increase in the activities of gametic glycolytic and Krebs cycle enzymes. The authors also concluded the likeliness that both carbohydrates and amino acids serve as energy sources for mature spermatozoa. At the same time, the results highlighted the low activities of lactate dehydrogenase and α-hydroxyacyl dehydrogenase, which indicates that gametes at all stages of development have limited abilities to produce lactic acid and oxidize fatty acids [132]. In contrast to this, it has been shown that mammalian spermatids are highly dependent on lactate [133].

For mammalian fertility, proper mitochondrial function is essential, and mitochondrial development and function vary greatly in different developmental steps [134]. Mitochondrial differentiation is initiated simultaneously with spermatogonial differentiation: cristae structure reorganization occurs in spermatocytes and spermatids, and further metabolic switches occur in sperms. Glycolytic energy production is characteristic of stem cells; meanwhile, dividing and differentiating cells use OXPHOS. The mature sperm uses both glycolysis for survival and OXPHOS for mobility and fertilization. ROS signaling is essential for both spermatogonia differentiation and sperm cell capacitation [7,18,49,135].

The disturbance of metabolic homeostasis is often reflected in mitochondrial morphology. We have to emphasize that there are no data on the transcription of nuclear-encoded mitochondrial genes in Drosophila after meiosis; therefore, the regulation of mitochondrial homeostasis could become challenging for differentiating spermatids [136,137]. The *bb8^ms^* mutant has a striking phenotype in this aspect: in the *bb8^ms^* mutant, the nebenkern is normal; however, megamitochondria form in the elongating spermatids [35,97]. The scale of these megamitochondria varies; however, the largest ones are comparable in size with a single round spermatid cell. The origin of these megamitochondria is not fully understood, but based on electron microscopic images, they could originate from the elongating mitochondrial derivatives. The abnormal mitochondrial structures in the testes are similar to those megamitochondria observed in cultured cells [138,139]. In spermatids, similar swollen mitochondria can be observed in the presence of ectopically expressed Aralar1 glutamate transporter [97]. In both cases, glutamate metabolism is affected, resulting in the accumulation of glutamate/glutamine in the mitochondria, which seems to be the reason for mitochondrial aberrations. Interestingly, mitochondrial abnormalities are characteristic of the tumor suppressor F-box protein, as well as Archipelago (Ago) mutants [99]. In the case of Ago, the mechanism affecting the mitochondrial development is unknown, but there is a known interolog interaction between Ago and Aralar1; however, Aralar1 upregulation was not tested on the Ago mutant background [140]. Moreover, Ago has potential connections to S6 kinase, Parkin, and hypoxia-induced gene expression [99]. Swollen mitochondrial structures can be observed also in ref(2)P mutants lacking the UBA domain [70]. The ref(2)P mutants are interesting in the aspect of the ref(2)P downstream position to the Pink1/Parkin pathway. In *pink1* mutants, a metabolic stress response results in the elevation of glutamate/glutamine levels [141]. We can assume a similar effect in ref(2)P UBA mutants, which could explain the formation of large vacuolated mitochondria and link the ref(2)P to the metabolic stress response. The human ortholog of ref(2)P, p62, was described in the cellular energy metabolism of differentiating neuronal cells: the absence of p62 promotes glycolytic energy metabolism [129]. Mitoferrin mutant *mfrn* exhibits a similar phenotype to the *bb8* mutant: large vacuolated vesicles are observable on phase-contrast micrographs, and although these structures were not tested with fluorescent dyes, the mitochondrial abnormalities were still visible on EM cross sections [75]. Other iron metabolism-related genes such as *fh* and *Fer3HCH* show higher expression in the testes, emphasizing the importance of iron metabolism during spermatogenesis [75]. If we try to address the observed phenotypes of mitoferrin deficiency, we might conclude that the lack of the transporter results in iron shortage and hinders the enzymatic activity of the mitochondrial iron–sulfur aconitase (mAcon1, mAcon2) enzymes or the proper function of the heme group containing catalases. Cytosolic aconitase has a proven effect on glutamate level regulation [142,143], which makes aconitases possible candidates for the observed mitochondrial swelling phenotype in the mitoferrin mutants. Catalases are present in the paracrystalline material of the major derivatives, which could explain the paracrystalline abnormalities in *mfrn* mutants [36,75]. Catalases decompose hydrogen peroxide, thus protecting the mitochondria against the toxic effects of ROS. Interestingly, the role of alpha-ketoglutarate, the metabolite of Bb8, was shown to defend against ROS with H_2_O_2_ scavenging [144]. Considering the effect of iron metabolism on Pink1 phenotypes, iron metabolism in testes still poses many interesting questions [74,145].

Metabolic mitochondrial abnormalities could occur as a consequence of the disturbance of nucleoside metabolism. Concentrative nucleoside transporter 1 (CNT1) is a membrane transporter that exhibits a predicted nucleoside sodium symporter activity. In CNT1 mutants, the development of mitochondrial derivatives is disturbed [146].

## 8. The Effect of Systemic Metabolism on Spermatogenesis

Systemic metabolism, nutrition, and communication between organs could impact germ cell development; although, in many cases, their effects converge into the mitochondria and mitochondrial metabolism.

In Drosophila males, the testes have a connection to the gut, where the testes induce the differentiation of a specific portion of the intestine through the Jak/Stat signaling pathway. The male-type intestine transfers citrate to the testis, which enhances the sperm production [147]. Interestingly, this citrate import does not serve primarily as an energy source, rather it is required for proteome stability. The excess citrate can be utilized to produce acetyl-coenzyme A, which promotes NatB-dependent N-terminal protein acetylation. This acetylation masks proteins from ubiquitin–proteasome degradation [128].

Dietary sugar also has an impact on Drosophila spermatogenesis. An increased amount of sugar results in shifts in mitochondrial activity, ROS production, and the expression of small RNAs. It has been suggested that this response to high dietary sugar might be an evolutionary conserved mechanism [148].

Spermatogenesis is closely linked to dietary factors in mammals as well. A sheep model of excessive-energy diet-induced metabolic syndrome highlighted the importance of metabolic influences on spermatogenesis and the related microbiome [149].

## 9. Mitochondrial Inheritance and Mitochondrial DNA

For proper sustainable mitochondrial function, the mitochondrial genome is essential. Interestingly, the mitochondrial genome’s maintenance in the germline heavily relies on mitochondrially targeted topoisomerase 3 alpha (Top3α) in Drosophila [150]. In testes, the absence of Top3α results in GSC loss.

In animals, mitochondria are maternally inherited cell organelles, and there are multiple mechanisms which could contribute to this uniparental inheritance. Both oocytes and sperms need high-quality mitochondria, as in oocytes, they need to be inherited to the next generation, while in sperm, the mitochondria are required to cover the high energy demand of movement. In active sperms, there is an increased risk of mitochondrial damage which cannot be addressed in the specialized cells, and that is not favorable for passing down to the next generation.

To prevent the inheritance of paternal mitochondria, the mitochondrial derivatives and sperm components are eliminated after fertilization by endocytic and autophagic processes in the embryo [104,151]. Despite this, it seems that there is an additional process during Drosophila spermatogenesis that actively eliminates the paternal mtDNA. The EndoG mitochondrial endonuclease is necessary for the elimination of mtDNA [152]. EndoG has five paralogs that show testis-specific transcript enrichment; however, Tengl1, Tengl2, Tengl3, and Tengl4 do not participate in mtDNA elimination, and CG12917 was not studied [152]. Surprisingly, the mtDNA polymerase (Pol γ-α), tamas (tam), also plays a role in mtDNA elimination to ensure uniparental inheritance [153]. Tam does not participate in mtDNA elimination with its 3′ exonuclease activity; it forms a larger protein complex and most probably acts as a regulator in the process [153].

The individualization mechanism also eliminates some of the mtDNA [152]. The mtDNA elimination during individualization seems to be an effect of the actin cones’ movement, as it trims mitochondrial mass [112]. Considering this, it is easy to hypothesize that the mtDNA, which has spatial requirements, becomes caught in the spherical mitochondrial structures, which are sheared by the individualization complex. Furthermore, we cannot rule out that mtDNA degradation might be a secondary effect of the lack of factors that maintain mtDNA integrity in these specialized cells.

Despite the above-mentioned mechanisms, it is common for a leakage of paternal mtDNA inheritance to occur in other Drosophila species, which has an even increased probability in hybrids [154,155,156].

## 10. Similarities and Differences between Drosophila and Mammalian Spermatogenesis in the Aspect of Mitochondrial Organization and Function

Despite insect and mammalian spermatogenesis exhibiting striking similarities, there are notable differences. Nevertheless, the process of spermatogenesis produces motile mature spermatozoa, with specialized mitochondria. The initial steps of germ cell differentiation are very similar: stem cells rely on glycolysis and metabolic changes through the promotion of OXPHOS to drive the differentiation of spermatogonia [16,157]. In both mammals and insects, mitochondria are dynamically reorganized in multiple consecutive steps during spermatogenesis. In Drosophila, this involves the formation of nebenkerns, and subsequently the formation of mitochondrial derivatives [10]. In mammals, in meiotic spermatocytes, the mitochondria also show dynamic reorganization, fusion, and fission during divisions, but after meiosis, they undergo fission [8]. This is required for proper mitochondrial sheath formation, and most probably for removing the excess or malfunctioning mitochondria to residual bodies. Whether the mechanism behind the elongation of mitochondria present in the midpiece involves mitochondrial fusion is still debated [49,50]. In humans, morphological changes in the mitochondria are less extreme compared to Drosophila, but they involve cristae reorganization and differentiation in the midpiece. During mammalian spermatogenesis, meiosis is damaged by the lack of mitofusin; the midpiece of the sperm is abnormal, and ultimately, there is a failure of the metabolic activity [158]. These results show the importance of mitofusins in promoting OXPHOS, leading to a metabolic shift during spermatogonial differentiation, mitochondrial remodeling, and elongation. Meanwhile, fission also plays a role in spermatid maturation in mammals, contrary to Drosophila spermatids, where mitochondrial mass is not fragmented [50]. Alongside structural reorganization, metabolic reorganization also happens. In mammals, this phenomenon is intensively studied; meanwhile, our knowledge on metabolism in insects is more limited.

In Drosophila, the mitochondrial derivatives in elongating spermatids and spermatozoa function as structural elements, as they are necessary for elongation and give structural rigidity through paracrystalline material accumulation [9,10]. In mammalian sperm, the mitochondria also serve a mechanical function by tightly coiling around the axoneme in the midpiece. This compact coiling is made possible by the presence of disulfide bonds within the mitochondrial sheath, which provide stability [2,103]. Structural abnormalities in the mitochondrial sheath often lead to abnormal sperms [50,104,105,106,107,108].

Despite similarities, there are undeniably large differences as well. Female insects store sperm for a longer time, and in mammals, the sperm travel larger distances. Insects produce fewer sperm, yet more of them fertilize eggs. These strategies have resulted in different evolutionary consequences. One of these consequences is the extreme length of the Drosophila sperms, and another could be the high motility and capacitation of mammalian sperm [159].

Mammalian spermatogenesis is considerably more complex. In Drosophila, the role of cyst cells is less well understood; meanwhile, Sertoli cells in humans are intensively studied. Sertoli and Leydig cells play an essential role in regulating the spermatogenic process. Sertoli cells make it possible to take the metabolic burden from the developing germ cells and feed them with specific metabolites, mainly pyruvate and lactate. We do not know if a similar feature is characteristic of the cyst cells of Drosophila. Nevertheless, in Drosophila, the developing germ cells are more autonomous than their human counterpart, as isolated cysts can be cultured in cell culture media [9]. Metabolic specialization is a general phenomenon, and it occurs in both systems. In Drosophila, a high number of testis-specific genes, including a variety of testis-specific metabolic enzymes [83], are described; however, in humans, testis-specific genes are not that abundant, but testis-enriched genes are common [160,161].

The metabolic changes are also different. Both the insect and mammalian spermatogenesis processes rely on carbohydrate metabolism, but Drosophila spermatids are not lactate-dependent, whereas human spermatids are. Mammalian mitochondrial differentiation starts with the initial differentiation of spermatogonia, and cristae structure reorganization occurs in spermatocytes and also in spermatids; moreover, further metabolic switches occur in sperms. In mammals, glycolytic energy production is a characteristic of stem cells, while dividing and differentiating cells use OXPHOS. The mature sperm uses both glycolysis for survival and OXPHOS for mobility and fertilization. ROS signaling is essential for both spermatogonia differentiation and sperm cell capacitation [7,18,49,135]. Glycolytic energy production in stem cells is a conserved feature; the role of ROS and OXPHOS is less characterized in Drosophila. Capacitation is not characteristic of Drosophila spermatozoa, but bidirectional movement is [162,163].

## 11. Open Questions

We have a greater insight into the early development, and mitochondrial function in the differentiation of spermatogonia. However, there is limited knowledge on specialized cristae structure formation: what are the molecular components of it, are there testis-specific components of it, how does it participate in OXPHOS, and is there an additional function we do not know about?

The Pink1/Parkin pathway’s involvement in mitochondrial dynamics is well documented, yet a more thorough examination is necessary to clarify the intricate regulation and molecular elements located both upstream and downstream of the pathway in Drosophila spermatogenesis.

Further questions arose as we investigated the post-meiotic stages, when mitochondria-associated testis-specific transcripts are enriched. What is the rate of OXPHOS during elongation, and individualization? Are there more proteins with moonlight functions in the late stages of spermatogenesis? Further experiments are needed to address these questions.

Another interesting question concerns the conservation of mitochondrial function and structure in different species. Are there unidentified processes that are common in most organisms? Regarding this, glutamate metabolism holds some interesting conundrums. Monosodium glutamate (MSG), a food additive, has been proven to cause male fertility problems in rodents [164,165,166,167]. Similar problems were not observed in humans [168]. The reason behind this might be the presence of an additional glutamate dehydrogenase enzyme variant in humans. The main specialty of this enzyme, GLUD2, is its ability to catabolize glutamate in the presence of GTP [169]. Interestingly, GLUD2 has similar features to Drosophila Bb8 glutamate dehydrogenase [97]. The effects of MSG intoxication in rodents are weaker when selenium is provided [170,171]. Similarly, selenium exhibits a neuroprotective function in glutamate-induced cytotoxicity in HT22 cells [172]. Selenium also plays a role in the regulation of Drosophila glutamate metabolism [138]. In bacteria, selenium is known to be associated with glutamate-accepting tRNA [173]. These findings raise the question of selenium’s conserved role in the regulation of glutamate metabolism and mitochondrial function.

## 12. Conclusions

Mitochondrial differentiation is highly dynamic and crucial during spermatogenesis. The role of mitochondria varies significantly, as they are involved in ROS signaling, act as a structural component, and serve as the powerhouse of cells. With each passing day, our understanding of these versatile organelles improves; however, there is still much to uncover. We have a general structural overview of mitochondrial organization in Drosophila, but there are still gaps in our knowledge regarding the finer structural reorganizations. Electron tomography stands as a formidable instrument for scrutinizing mitochondrial structures. The prospect of unveiling the three-dimensional ultrastructure of mitochondria at distinct stages of Drosophila spermatogenesis adds a compelling dimension to the exploration of mitochondrial reorganization.

The energetics and possible metabolic shifts during the later phases of spermatogenesis have not been comprehensively discovered. The diverse functionalities of mitochondria with distinct structures remain incompletely understood. How the classical mitochondrial function undergoes alteration within the nebenkern or between major and minor mitochondrial derivatives is a subject yet to be fully elucidated. The role of the testis-enriched genes is also an interesting field, where molecular specialization and evolutionary aspects are intriguing.

This review primarily centered on the mitochondrial alterations, roles, and functions occurring during Drosophila spermatogenesis, while also providing a glimpse into their mammalian counterparts. We presented an exhaustive overview of the morphological transformations in mitochondria observed within the Drosophila testes. Additionally, we posed a series of inquiries exploring intriguing evolutionary, functional, and structural dimensions of mitochondrial differentiation across diverse developmental stages.

We posit that the exploration of specialized mitochondria within the Drosophila male germline harbors significant potential for future discoveries, given its substantial contribution to advancing our comprehension of mitochondrial intricacies.
